# The prognostic influence of body mass index, resting energy expenditure and fasting blood glucose on postoperative patients with esophageal cancer

**DOI:** 10.1186/s12876-016-0549-6

**Published:** 2016-12-21

**Authors:** Ning Wu, Yongjun Zhu, Dhruba Kadel, Liewen Pang, Gang Chen, Zhiming Chen

**Affiliations:** 1 Department of Cardio-thoracic Surgery, HuaShan Hospital of Fudan University, Shanghai, 200040 People’s Republic of China; 2 Department of General Surgery, HuaShan Hospital of Fudan University, Shanghai, 200040 China

**Keywords:** Esophageal cancer, Body mass index, Resting energy expenditure, Fasting blood glucose, Prognosis

## Abstract

**Background:**

Body mass index (BMI), resting energy expenditure (REE) and fasting blood glucose (FBG) are major preoperative assessments of patients’ nutrition and metabolic state. The relations and effects of these indices on esophageal cancer patients’ postoperative short-term and long-term outcomes remain controversial and unclear. We aimed to study the impact of BMI, REE and FBG in esophageal cancer patients undergoing esophagectomy.

**Methods:**

Three hundred and six esophageal cancer patients who underwent esophagectomy were observed retrospectively. Clinical characteristics, postoperative complications and survival analysis were compared among different BMI, REE and FBG groups.

**Results:**

There were significant linear relationships between REE, BMI and FBG indices, patients with low BMI tended to have low REE (*p* < 0.001) and low FBG (*p* = 0.003). No significant difference was found in case of mortality and postoperative complications among different groups. Low BMI (*X* ^2^ = 6.141, *p* = 0.046), REE (*X* ^2^ = 6.630, *p* = 0.010) and FBG (*X* ^2^ = 5.379, *p* = 0.020) were related to poor survival. FBG ≤90 mg/dL was independently associated with poor survival (HR = 0.695; 95 % CI 0.489–0.987, *p* = 0.042). BMI and REE came to be stronger prognostic factors on lymph node-negative patients and proved to be independent prognostic indicators (HR = 0.540; 95 % CI 0.304–0.959, *p* = 0.035 and HR = 0.457; 95 % CI 0.216–0.967, *p* = 0.041, respectively).

**Conclusions:**

BMI, REE and FBG are important prognostic factors in patients with esophageal cancer undergoing esophagectomy and preoperative evaluation of these indices help to determine the prognosis in these patients.

## Background

Esophageal cancer (EC) is the eighth most common cancer and the sixth leading cause of cancer mortality worldwide [1]. Esophageal adencarcinoma (EAC) and esophageal squamous cell carcinoma (ESCC) are the most frequent histological subtypes. ESCC is the dominant histological subtype in china [2]. The potential prognostic indicators of esophageal cancer include histological variants (histological grading, differentiation, invasion depth and classification of lymph node metastasis) and nutrition or inflammation based prognostic factors (total lymphocyte counts, neutrophil to lymphocyte ratio (NLR), serum albumin and so on). BMI, REE and FBG, which are widely used to assess preoperative nutritional and metabolic status, have also been described to be prognostic predictors in several tumors [3].

Previous studies reported that high BMI was associated with increased risk of EAC while low BMI with ESCC [4]. High BMI in surgical patients is thought to be associated with increased comorbidities and postoperative complications, but the influence of high BMI on survival in patients undergoing esophagectomy is controversial. Hayashi found patients with high BMI showed better overall survival (OS) and disease-free survival (DFS) because of the early clinical diagnosis [5]. In contrast to this, Yoon pointed out that high BMI was independently associated with two-fold worsening of DFS, and OS after surgery for EAC [6]. Blom concluded that BMI had no prognostic value on short-term and long-term outcomes [7]. Compared with the previous studies, the mean BMI in Western populations was higher and had a greater incidence rate of EAC whereas the mean BMI in Chinese population was found to be lower with predominant ESCC. The effect of low BMI on postoperative complications and long-term survival remains unclear. So our study assessed the relationship between BMI and postoperative complications and long-term survival in Chinese population.

Disorder of energy metabolism is a common phenomenon in cancer patients. The energy metabolic status among different cancers may not be the same. Many researchers assessed the resting energy expenditure on cancer patients and no unanimous conclusions have been drawn. In this study, patients’ preoperative REE was an estimated variable, and calculated by the Mifflin-St. Jeor equation [8]. REE is the sum of the metabolic activities of internal body and can reflect patient’s physiques and muscle volumes, and REE per kg total body weight (REE/kg) may reflect the diffusion and metabolic rate of muscle more accurately. So both REE and REE/kg were used to explore its effects on short-term and long-term outcomes on esophageal cancer patients.

Epidemiologic evidence suggests that elevated blood glucose is associated with many forms of cancer [9]. However, opinions toward the association between elevated blood glucose and outcomes are mixed. Elevated blood glucose may promote cancer progression and lead to poor outcomes via pathways mediated high levels of insulin and insulin-like growth factor [10]. On the other hand, some studies proposed that diabetes-related microvessel changes might play a protective role against “neoplastic” cell metastasis in cancer patients and enhance cancer prognosis [11]. For EC, the relationship between the circulating glucose levels and prognosis has never been reported. To examine effects of fasting glucose levels at time of cancer diagnosis on postoperative outcomes is another aim of this study.

So the purpose of the study is to investigate the prognostic value using combined assessment of BMI, REE and FBG in esophageal cancer patients.

## Methods

### Patients

Patients who underwent esophagectomy and lymph node dissections during Sep 1, 2003 to Dec 31, 2008 were observed. Twenty-three patients who had palliative or R1/R2 resections were excluded. Patients who had histology other than SCC or AC (one lymphoma, one melanoma, one neuroendocrine carcinoma, one stromal tumor and two small cell carcinoma) were excluded. Patients who received neoadjuvant therapy were also excluded. Three hundred and six patients with histologic documentation of AC or SCC were included. All patients provided written consent and this retrospective study was approved by the Institutional Review Board of Huashan Hospital, Fudan University.

### Clinical data

Preoperative staging was performed in all patients by means of barium meal test, fibro-gastroscopy, computed tomography of the chest and abdomen and ultrasound of the neck and retroperitoneal lymph nodes. Some patients also received whole body positron emission tomography (PET)-CT scanning. All patients were assessed for physiological ability to undergo esophagectomy. These evaluations included pulmonary function test, cardiac function test and nutritional assessment.

One hundred and eighty-six patients with tumors in middle or lower thoracic esophagus and no evidence of lymph node involvement in the superior mediastinum or neck region received transthoracic esophagectomy via left thoracotomy which also included a two-field lymphadenectomy; 53 patients with tumors in the middle or upper thoracic esophagus and possible lymph node metastasis in the superior mediastinum or neck region received McKeown 3-hole esophagectomy with three-field lymphadenectomy; 67 patients with tumors in the esophagogastric junction received esophagogastrectomy through median laparotomy.

TNM staging was performed according to the AJCC 7th edition guidelines. Patients were followed up every 6 months for the first 3 years and then annually. Survival time was measured as the time from the date of surgery to the date of death or the latest follow-up time. Endoscopy, CT, PET-CT and Radionuclide bone scan were performed if recurrent or metastatic disease was suspected.

Patients’ weight and height were measured at their first hospitalization. BMI was calculated as weight in kilograms divided by height in meters squared.

REE was calculated by Mifflin-St. Jeor equation. The applied technique to calculate BMI is as follows: for males = 10 * weight (kg) + 6.25 * height (cm) - 5 * age (y) + 5; for females = 10 * weight (kg) + 6.25 * height (cm) - 5 * age (y) - 161. Both REE and REE/kg were calculated.

The serum FBG concentration of patients was measured in the morning during the first hospitalization by hexokinase method after fasting for 10 h. Serum albumin was measured by means of the bromocresol purple method using automated equipments, and all patients were screened and excluded from acute and chronic liver disease. The complete blood count test (leukocyte, neutrophil, lymphocyte, monocyte, eosinophil, basophil, platelet counts, and hemoglobin) was carried out by an automated haematology analyser within one week prior to surgery. NLR was calculated as the ratio of neutrophils to lymphocytes in peripheral blood. These reagents and equipments were convenience-validated and standardized in our central clinical laboratory.

The 6-month preoperative weight loss was also measured and patients were divided into three categories: No/Little (loss of 0 to 5 % body weight), Middle (loss of 5 to 10 % body weight) and Large (loss more than 10 % body weight).

During the first few days after surgery, patients were treated with total parenteral nutrition. An initial dose of 5–10 kcal.day^−1.^kg^−1^ of enteral nutrition was supplied via a duodenum or jejunum feeding tube from the 2nd or 3rd postoperative day and gradually increased to the full dose of 25–30 kcal.day^−1.^kg^−1^. Some patients with low BMI or low serum albumin levels were treated with full dose of enteral nutrition from one week before surgery to the tenth day after surgery.

### Statistical analysis

All statistical analysis was performed with SPSS 16.0. Spearman rank order correlation coefficient (Spearman’s rho) for nonparametric data was used. Univariate analysis of survival was performed using Kaplan-Meier method and log-rank test to estimate the prognostic value. Multivariate analysis of survival was performed using Cox-regression model to estimate hazard ratios (HRs) with 95 % confidence intervals (CI) and identify independent prognostic factors. The level of significance was set to *p* < 0.05.

## Results

### Patient characteristics

The clinical characteristics and 5-years survival rate are summarized in Table 1. The median follow-up time was 37 months. The overall 1-, 3- and 5-year survival rate was 86.6, 60.8 and 47.1 % respectively.

**Table 1:** Patient characteristics and univariate analysis

Factors		N	5-year survival (%)	*p*
Age, year	≤65	210	53.3	**0.003**
	>65	96	34.6	
Sex	Male	235	44.0	**0.039**
	Female	71	57.8	
Histology	AC	98	49.7	0.590
	SCC	208	45.7	
Surgical type	Transthoracic	186	47.0	0.701
	McKeown	53	40.4	
	Transabdominal	67	51.2	
Differentiation	Well	38	61.9	**0.000**
	Moderately	173	55.1	
	Poorly	95	24.9	
T stage	Tis/T1	31	82.2	**0.000**
	T2	65	62.1	
	T3	159	43.3	
	T4a	51	17.1	
N stage	N0	156	67.5	**0.000**
	N1	73	38.6	
	N2	52	14.2	
	N3-4	25	9.5	
TNM stage	0-I	42	80.5	**0.000**
	II	118	66.5	
	III	146	21.3	
Weight lost	No/Little	203	46.5	0.555
	Middle	50	56.8	
	Large	53	41.7	
Adjuvant Chemoradiation	Yes	78	41.7	0.187
	No	228	48.8	
Albumin	<35 g/l	36	26.7	**0.000**
	35–40 g/l	132	41.6	
	>40 g/l	138	57.8	
Lymphocyte	<1.1*10^9^	42	48.7	0.780
	1.1–3.2*10^9^	228	47.5	
	>3.2*10^9^	36	42.6	
NLR	<5	289	47.1	0.787
	≥5	17	47.1	
REE/kg	<23.2	245	50.4	**0.024**
	≥23.2	61	35.2	
REE	Low	154	40.4	**0.010**
	High	152	55.3	
FBG	Low	108	35.0	**0.020**
	High	198	51.7	
BMI	Low	81	36.8	**0.046**
	Normal	186	49.1	
	High	39	62.8	

*CI* confidence interval, *OR* odds ratio

The results were in bold, if the 95 % CI excluded 1 or *p*<0.05

The BMI distribution was as follows: low (<20 kg/m^2^), *n* = 81 (26.5 %); normal (20–25 kg/m^2^), *n* = 186 (60.8 %) and high (>25 kg/m^2^), *n* = 39 (12.7 %). The median REE was 1387.5 kcal day^−1^ for male and 1064.0 kcal day^−1^ for female where we defined the median REE as the cutoff point and stratified patients into low REE group (male < 1387.5 and female <1064.0), *n* = 154 (50.3 %) and high REE group (male ≥1387.5 and female ≥1064.0), *n* = 152 (49.7 %). The median REE/kg was 21.49 kcal.day^−1.^kg^−1^. Compared with 245 (80.1 %) patients with REE/kg < 23.2, 61 (19.9 %) patients with REE/kg ≥23.2 had significant worse survival, so 23.2 kcal.day^−1.^kg^−1^ was set as the cutoff point. We divided the patients into two groups according to serum FBG concentration where 108 patients (35.3 %) with low FBG (≤90 mg/dL) and 198 patients (64.7 %) with high FBG (>90 mg/dL).

### Patient characteristics by BMI, REE, REE/kg and FBG

As shown in Table 2. Spearman’s rho indicated that patients with age > 65 years (*p* < 0.001) and advanced T-stage (*p* = 0.040) were more likely to fall in the low REE class. Smoking patients were more likely to be associated with low BMI class (*p* = 0.013), low FBG class (*p* = 0.039) but high REE/kg class (*p* = 0.001).

**Table 2:** Associations among characteristics, BMI, REE, REE/kg and FBG

Factors	BMI, kg/m^2^				REE, kcal day^−1^			REE/kg, kcal day^−1^			FBG, mg/dL		
	<20	20–25	>25	***p***	Low	High	***p***	Low	High	***p***	≤90	>90	***p***
	81	186	39		154	152		245	61		*108*	198	
Age													
≤ 65	50	134	26	0.332	87	123	**0.000**	157	53	**0.001**	73	137	0.774
> 65	31	52	13		67	29		88	8		35	61	
Sex													
Male	65	141	29	0.408	118	117	0.942	174	61	**0.000**	88	147	0.153
Female	16	45	10		36	35		71	0		20	51	
Histology													
SCC	54	132	22	0.561	103	105	0.682	165	43	0.639	81	127	0.052
AC	27	54	17		51	47		80	18		27	71	
Differentiation													
Well	7	28	3	0.869	19	19	0.507	31	7	0.552	14	24	0.557
Moderately	50	98	25		91	82		140	33		63	110	
Poorly	24	60	11		44	51		74	21		31	64	
T stage													
Tis/T1	8	19	4	0.172	12	19	**0.040**	27	4	0.124	12	19	0.499
T2	14	42	9		30	35		54	11		22	43	
T3	42	93	24		81	78		126	33		50	109	
T4a	17	32	2		31	20		38	13		24	27	
N stage													
N0	36	98	22	0.241	73	83	0.476	131	25	0.262	54	102	0.551
N1	20	44	9		42	31		55	18		28	45	
N2	20	27	5		26	26		38	14		22	30	
N3-4	5	17	3		13	12		21	4		4	21	
Bad habits													
Smoking	51	86	17		77	77	0.909	112	42	**0.001**	63	91	**0.039**
Drinking	26	60	11		46	51	0.490	74	23	0.261	38	59	0.355
Comorbidities													
Highblood	12	35	12	0.063	27	32	0.437	50	9	0.318	16	43	0.145
Copd	12	18	5	0.478	24	11	**0.022**	29	6	0.662	13	22	0.809
Cardiovascular disease	2	5	0	0.551	6	1	0.058	5	2	0.564	7	0	**0.000**
Arrhythmia	7	16	8	0.129	14	17	0.546	27	4	0.303	14	17	0.227
Diabetes	15	41	18	**0.006**	35	39	0.551	66	8	0.024	0	74	**0.000**
**Mortality**	1	5	1	0.970	6	1	0.058	6	1	0.706	3	4	0.673
Postoperative complications													
Fistula	6	6	3	0.083	12	3	0.182	9	6	0.259	6	9	0.825
Sepsis	9	6	6	0.208	15	6	0.260	12	9	0.125	9	12	0.673
Pneumonia	7	11	8	0.615	16	10	0.892	20	6	0.676	8	18	0.233
Respiratory insufficiency	6	9	6	0.846	14	7	0.692	18	3	0.504	7	14	0.122
Arrhythmia	14	33	8	0.661	30	25	0.419	45	10	0.720	18	37	0.491
Cardiac insufficiency	8	38	4	0.068	30	20	0.950	42	8	0.125	12	38	0.136
Albumin													
< 35 g/l	16	19	1	**0.001**	29	7	**0.000**	26	10	**0.009**	20	16	**0.000**
35–40 g/	38	78	16		68	64		99	33		55	77	
> 40 g/l	27	89	22		57	81		120	18		33	105	
Lymphocyte													
< 1.1*109	13	26	3	0.092	23	19	**0.031**	34	8	0.305	15	27	0.401
1.1–3.2	64	133	32		121	108		179	50		84	145	
> 3.2	4	27	4		10	25		32	3		9	26	
NLR													
< 5	73	179	37	0.135	144	145	0.473	233	56	0.316	101	188	0.603
≥ 5	8	7	2		10	7		12	5		7	10	
Weight lost													
No/Little	45	125	33	**0.001**	103	100	0.731	171	32	**0.006**	66	137	0.543
Middle	14	33	3		21	29		38	12		25	25	
Large	22	28	3		30	23		36	17		17	36	
FBG													
Low	37	64	7	**0.003**	65	43	**0.011**	79	29	**0.025**			
High	44	122	32		89	109		166	32				
REE													
Low	71	82	1	**0.000**				107	47	**0.000**			
High	10	104	38					138	14				
REE/kg													
Low	32	174	39	**0.000**									
High	49	12	0										

*CI* confidence interval, *OR* odds ratio

The results were in bold, if the 95 % CI excluded 1 or *p*<0.05

It was interesting to explore the relationship between REE and REE/kg. Both high REE and low REE/kg patients were found to have better survival, which was consistent with the spearman correlation analysis that found patients with high REE tended to have lower REE/kg (*p* < 0.001).

There were significant linear relations between BMI, REE, and FBG, patients with low BMI tended to have low REE (*p* < 0.001) and low FBG (*p* = 0.003). The trend of a linear association between REE and FBG could also be seen (*p* = 0.011).

There were close relations between REE/kg and weight lost or BMI, patients with high REE/kg have been found to lose more weight (*p* = 0.006) and have lower BMI (*p* < 0.001).

Low FBG was more likely to be seen in patients with high REE/kg (*p* = 0.025) and low albumin (*p* < 0.001).

### Comorbidities, postoperative mortality and postoperative complications

Preoperative comorbidities, mortality and postoperative complications among different groups of BMI, REE, REE/kg and FBG were presented in Table 2. COPD appeared to be more frequent in low REE patients (*p* = 0.022), and diabetes was more common in high BMI patients (*p* = 0.006). All the seven patients with cardiovascular disease belonged to low FBG class (*p* < 0.001). Observing the short-term outcomes, no significant difference was found in postoperative mortality and major postoperative complications among different BMI, REE, REE/kg and FBG groups.

### Nutrition or inflammation-based prognostic factors

Univariate analysis of nutrition or inflammation-based prognostic factors found to be serum albumin (*p* < 0.001), but not Total lymphocyte counts (*p* = 0.780) or NLR (*p* = 0.787), which was positively related to survival. The spearman correlation analysis found higher serum albumin levels were observed in high BMI, REE and FBG but in low REE/kg groups.

### Univariate, multivariate and subgroup analysis

**Fig. 1:**
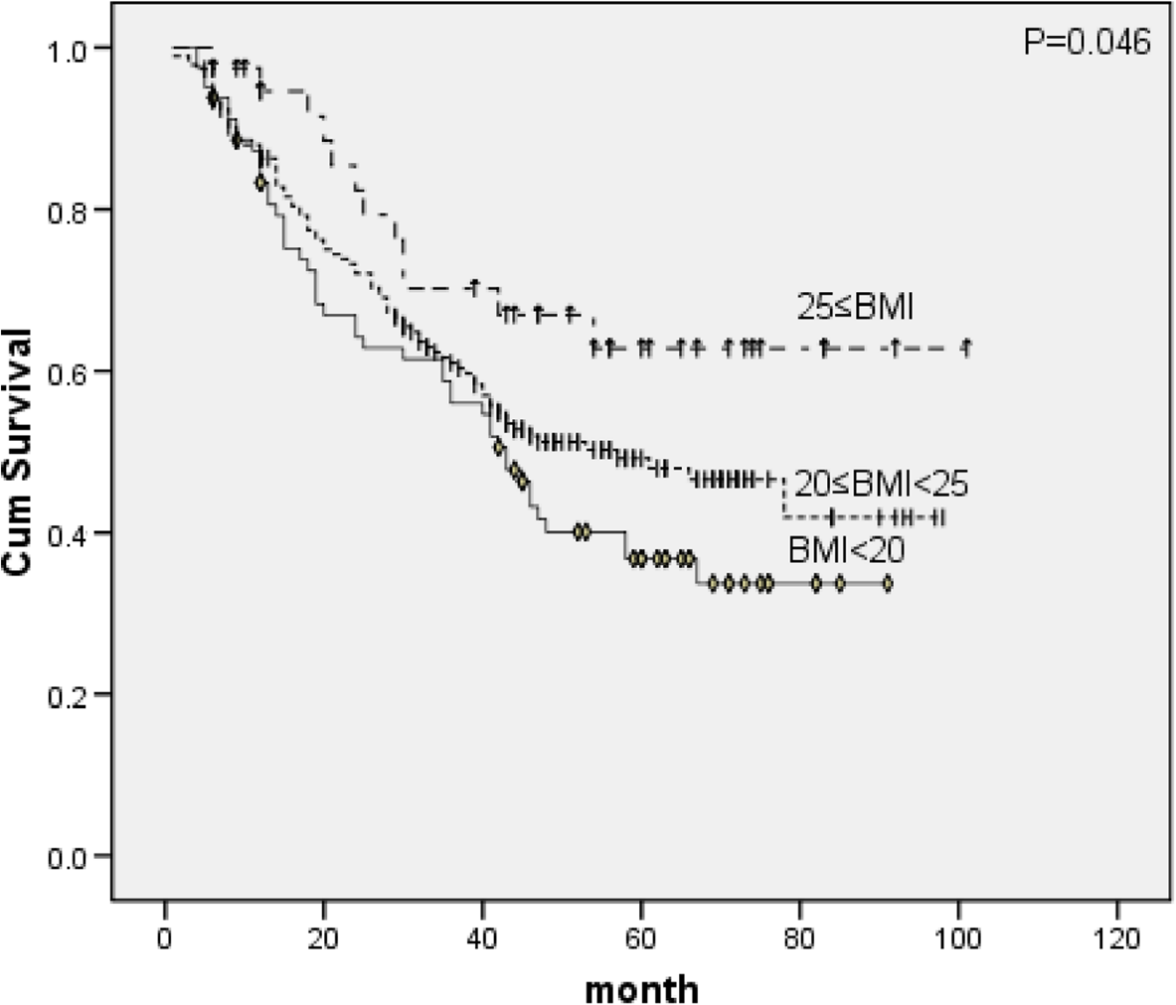
Survival curves of BMI classes

**Fig. 2:**
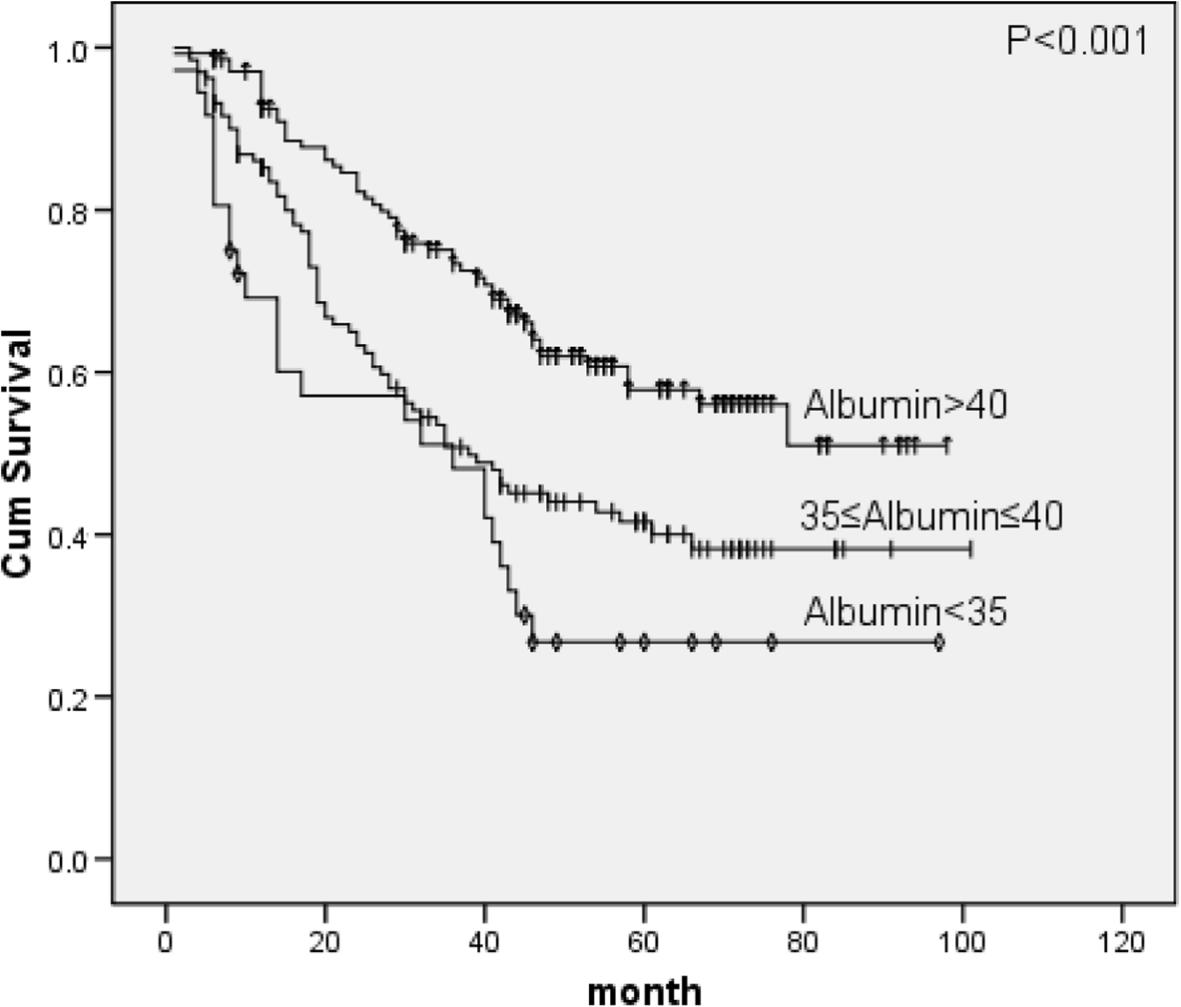
Survival curves of albumin classes

**Fig. 3:**
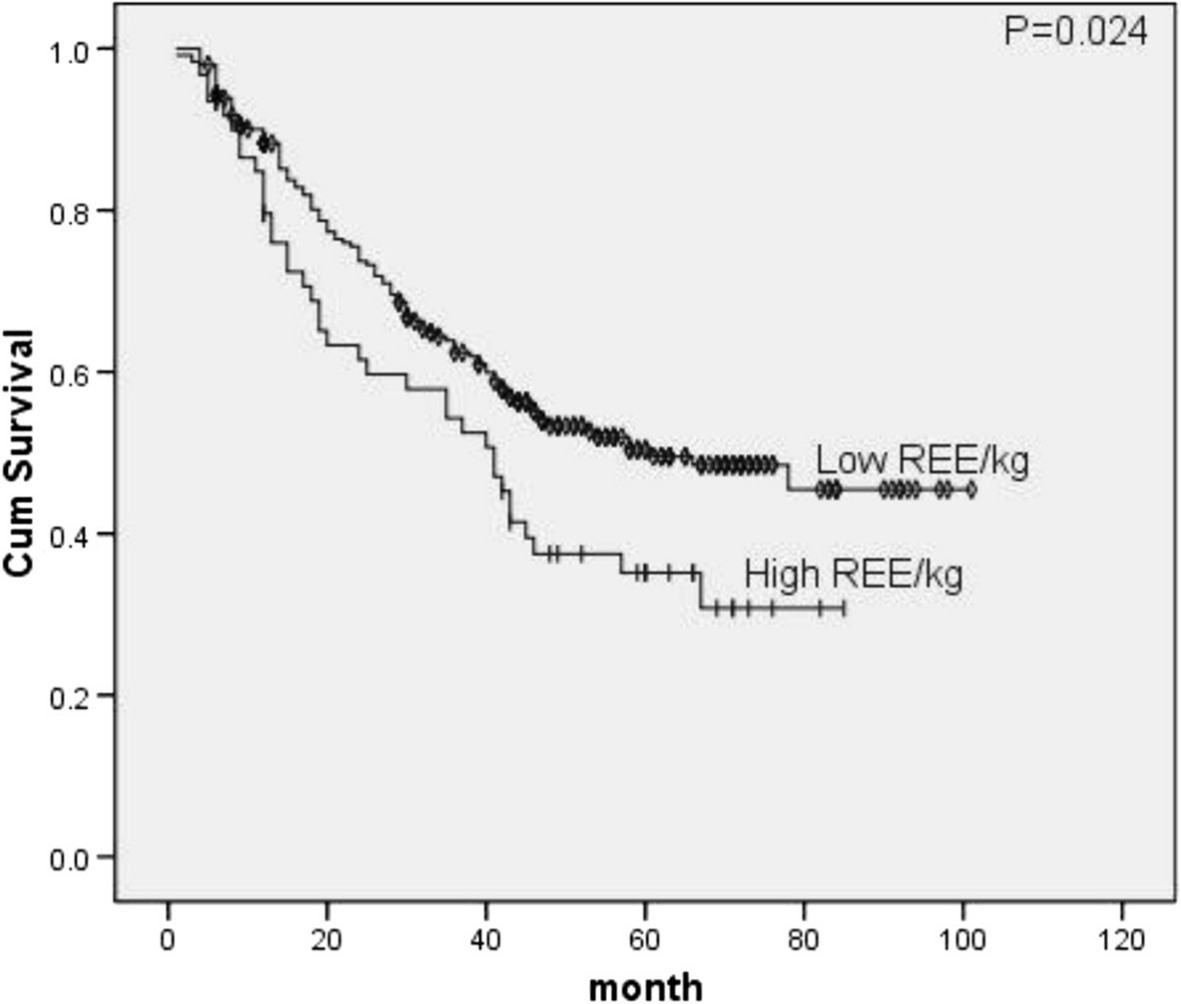
Survival curves of REE/kg classes

**Fig. 4:**
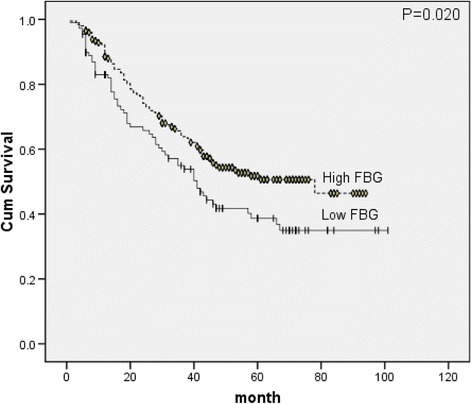
Survival curves of FBG classes

To evaluate the prognostic factors potentially related to survival, univariate analysis was applied (Table 1) and found no statistical associations of histologic subtype, surgical type, weight loss and adjuvant chemoradiation with OS. The prognostic factors were age, sex, differentiation, T-stage, N-stage, BMI (*X* ^2^ = 6.141, *p* = 0.046, Fig. 1), albumin (*X* ^2^ = 19.761, *p* < 0.001, Fig. 2), REE (*X* ^2^ = 6.630, *p* = 0.010), REE/kg (*X* ^2^ = 5.063, *p* = 0.024, Fig. 3) and FBG (*X* ^2^ = 5.379, *p* = 0.020, Fig. 4). Patients with low BMI, REE, FBG and high REE/kg had significant worse survival.

Variables significant (age, sex, differentiation, T-stage, N-stage, albumin, BMI, REE, REE/kg and FBG) in Univariate analysis were included in a multivariate analysis (Table 3), which did not show a significant association between BMI (HR = 0.945; 95 % CI 0.660–1.351, *p* = 0.755), REE (HR = 1.101; 95 % CI 0.718–1.688, *p* = 0.660) or REE/kg (HR = 1.164; 95 % CI 0.717–1.890, *p* = 0.540) and OS. Age, differentiation, T-stage, N-stage, albumin (HR = 0.757; 95 % CI 0.589–0.973, *p* = 0.030) and FBG (HR = 0.695; 95 % CI 0.489–0.987, *p* = 0.042) were independent prognostic factors.

**Table 3:** Multivariate prognostic analysis

Factors	All patients			N0 patients			N1-4 patients		
	*P*	OR	*95 % CI*	*P*	OR	*95 % CI*	*P*	OR	*95 % CI*
Age	**0.001**	1.844	1.279–2.661	0.286	1.417	0.747–2.689	**0.005**	1.982	1.228–3.200
Sex	0.229	0.757	0.481–1.191	**0.016**	0.309	0.119–0.803	0.465	0.818	0.478–1.401
Differentiation	**0.004**	1.560	1.154–2.107	0.761	0.921	0.542–1.564	**0.002**	1.885	1.267–2.805
T stage	**0.005**	1.463	1.125–1.903	**0.024**	1.611	1.065–2.436	**0.018**	1.528	1.076–2.168
N stage	**0.000**	1.610	1.366–1.898	-	-	-	0.056	1.317	0.992–1.749
BMI	0.755	0.945	0.660–1.351	**0.035**	0.540	0.304–0.959	0.789	0.939	0.594–1.485
REE	0.660	1.101	0.718–1.688	**0.041**	0.457	0.216–0.967	0.163	1.493	0.851–2.619
REE/kg	0.540	1.164	0.717–1.890	0.060	2.054	0.971–4.343	0.739	0.905	0.503–1.628
FBG	**0.042**	0.695	0.489–0.987	0.632	0.863	0.474–1.574	**0.043**	0.627	0.399–0.985
Albumin	**0.030**	0.757	0.589–0.973	0.863	0.960	0.605–1.524	0.198	0.819	0.604–1.110

*CI* confidence interval, *OR* odds ratio

The results were in bold, if the 95 % CI excluded 1 or *p*<0.05

In order to assess the impact of BMI and REE on different tumor stages, we divided patients into subgroups on the basis of N-stage (N0 vs. N1-4). Patients with higher BMI and REE had significantly better OS in subgroup of N0 (*X* ^2^ = 15.507 *p* < 0.001 and *X* ^2^ = 14.717, *p* < 0.001, respectively), but no statistical significance in subgroup of N1-4 (*X* ^2^ = 1.952, *P* = 0.377 and *X* ^2^ = 0.386, *p* = 0.534, respectively). Multivariate analysis of subgroups revealed BMI (HR = 0.540; 95 % CI 0.304–0.959, *p* = 0.035) and REE (HR = 0.457; 95 % CI 0.216–0.967, *p* = 0.041) were independent prognostic factors for N0 patients but not for N1-4 patients.

## Discussion

Our study identified that FBG level ≤90 mg/dL was independently associated with poor survival and also confirmed that advanced cancer stages remain the most powerful prognostic factors. In addition, we observed that BMI, REE and FBG were significant prognostic factors, but the prognostic value of BMI and REE is not the same between different lymph node metastasis statuses. For metastatic esophageal cancers, the most important prognostic factors were FBG, albumin and cancer staging including differentiation and invasion depth whereas BMI and REE did not significantly affect the OS. However, for non-metastatic esophageal cancers, BMI and REE were important risk factors and proved to be independent prognostic indicators.

Total lymphocyte counts, NLR and serum albumin were recognized as nutrition based or inflammation-based prognostic factors. Among the cancer patients, those with digestive tract malignancies were more likely to suffer from hypoalbuminaemia, which has been attributed to increased catabolism, obstruction of the digestive tract and the systemic inflammatory response [12]. The combined use of albumin and serum C-reactive protein were introduced by the Glasgow prognostic score [13, 14] and the combined use of albumin and lymphocyte counts have been mentioned by the Onodera’s prognostic nutritional index [15]. Hypoalbuminaemia has been shown to correlate with ideal body weight, weight loss, body cell mass and poor prognostic in cancer patients [16]. In our cohort, hypoalbuminaemia associated with weight loss, low REE and low FBG. Patients with serum albumin levels <35 g/l had the 5-year survival rate of 26.7 %, compared to 57.8 % in patients with serum albumin > 40 g/l. Serum albumin was proved to be an independent significant prognostic factor by multivariate analysis even after adjusting for potential confounding factors. To assess the impact of albumin on different tumor stages, we divided patients into subgroups by N-stage (N0 vs. N1-4), and found hypoalbuminaemia as a worse survival factor in both N0 group (*X* ^2^ = 11.078, *p* = 0.004) and N1-4 group (*X* ^2^ = 7.236, *p* = 0.027). But the multivariate prognostic analysis for subgroups of N0 and N1-4 exhibited that serum albumin as a weaker prognostic value than other potential prognostic factors. It may be explained by the different clinical characteristics of each subgroup which may impact on the prognostic value.

As a particular nonspecific marker of systemic inflammation, an elevated NLR is hypothesized to be associated with poor survival in various solid tumors [17]. But for esophageal cancer, its effect on long-term outcome is still controversial [18, 19]. Most of previous studies did not find the positive predictive values of NLR in patients with esophageal cancer [20, 21]. In this study, there was no significant difference in survival as the NLR cutoff value taken as five because many previous literatures reported optimal cutoff value as five [22]. We also did not find the prognostic value of total lymphocyte counts in patients with esophageal cancer.

A few studies focused on the influence of BMI on postoperative outcomes in patients with EC and had shown contradictory results, varying from no differences in postoperative complications and mortality to a higher incidence of individual complications (such as respiratory complications and anastomotic leak) in high BMI patients [5–7]. In our study, we found no difference of the frequency of postoperative complications and mortality among different BMI patients. In addition, patients with a high BMI had a higher frequency of diabetes. Smoking patients appeared to have a lower BMI. Although smoking is the preventable cause for esophageal cancer, but it was not observed to be a poor prognostic factor in our finding.

Previous studies had revealed contradictory results regarding the association between BMI and long-term outcome in esophageal cancer patients [4–7, 23–25]. Most of these studies were conducted in western populations that have a high incidence rate of EAC, which occurs more frequently in patients with a high BMI. But the main type in Chinese populations is ESCC (67.9 % in our data). The population is prone to be lean (26.5 % with BMI < 20 and only 12.7 % with BMI > 25 in our data) compared to Western populations. Based on our data, compared with normal patients, we found a significantly worse OS in low BMI patients and a significantly better OS in high BMI patients on a univariate analysis. The prognostic effect of BMI seems to be more valuable on lymph node-negative patients than lymph node-positive patients and proved to be independent prognostic indicators.

High blood glucose level makes patients more susceptible to certain postoperative complications such as surgical site infections, sepsis, myocardial ischemia and so on [26]. But no similar studies were reported concerning esophageal cancer. Based on our date, FBG did not affect the risk of all-cause mortality and postoperative complications. Our study found low FBG level and hypoalbuminaemia are inter-related. In the class of albumin <35 g/l, only 44.4 % (16/36) patients had the FBG > 90 mg/dL, but in the class of albumin >40 g/l, almost 76.1 % (105/138) patients had the FBG > 90 mg/dL, and both hypoalbuminaemia and low FBG level were proved to be worse survival factors. It is easy to measure and monitor FBG or serum albumin in clinical research, so both FBG and serum albumin can easily be used for survival prognosis, and researchers can choose the appropriate method according to their laboratory conditions. To assess the impact of FBG on prognosis in patients with esophageal cancer, both the univariate and multivariate analysis proved that low FBG level (≤90 mg/dL) was independently associated with poor OS. These finding is consistent with previous studies which suggested that hyperglycemia related microvessel changes may have a protective effect against neoplastic cell spread and metastasis in patients with malignant tumors such as non-small cell lung cancer and so on [27, 28].

REE is believed to be elevated in several types of tumors, It has been hypothesized that increased energy expenditure may contribute to the development of malnutrition and weight loss [29]. Our study found patients with a low REE had significantly worse survival compared with high REE (5-year survival rate: 40.4 % vs 55.3 %, *p* = 0.010). In addition, there was no significant association of REE with postoperative complications and mortality. To our knowledge, it is the first demonstration of the prognostic value of estimated REE in postoperative patients with EC. Our study also observed a significant linear correlation between BMI and REE (*p* < 0.001), showing that patients with low BMI tended to have low REE. As the estimated REE largely depends on patients’ weight and height, the worse prognosis for low REE patients may reflect that these patients are under-nourished and are likely to have poor prognosis as a result. In addition, patients with low REE tended to be associated with advanced T-stages, so we concluded that the advanced tumor stages may be another reason to explain the low REE patients’ worse survival.

We also explore the relationship between REE and REE/kg. We found the effect of REE on patient survival was opposite to that of REE/kg. Low REE as well as high REE/kg was found to be a potential worse prognostic indicator. The existing hypothesis of REE is, the sum of the metabolic activities of internal organs, muscle, bone, and adipose tissue and can reflect patient’s physiques and muscle volumes, but REE/kg may reflect the diffusion, consumption and metabolic rate of muscle. So patients with high REE/kg have been found to lose more weight, have lower BMI and have a worse survival, this finding is consistent with the hypothesis.

In this research, low FBG was significantly associated with low BMI, albumin and REE, a potential explanation might be proposed. Individuals with esophageal cancer commonly experience metabolic abnormalities. The abnormal state of insulin (hyperinsulinemia or insulin resistance) is one cause, and it promotes cancer growth and progression through its effects on the insulin and insulin-like growth factor pathways [30]. Meanwhile, diabetic microangiopathy render the vascular basal membrane less digestible by tumor cells, which may play a role in impeding neoplastic cell spread and metastasis.

Following are the main limitations in our study. First, this is a retrospective study and the sample size is not large. Second, all-cause mortality is used instead of disease-specific deaths, the latter is difficult to confirm. Third, we only chose R0 patients, so our results may not be suitable for all patients.

## Conclusion

In conclusion, it is the first article to explore the prognostic significance of BMI, REE and FBG among patients with EC. FBG level ≤90 mg/dL was independently associated with poor survival for all patients. BMI and REE were important prognostic factors and the value was significant on lymph node negative patients. Therefore it is advisable that the combined assessment of BMI, REE and FBG can be used for a better preoperative assessment and prognostic evaluation in esophageal cancer patients.

## Abbreviations

AC: Adencarcinoma
BMI: Body mass index
CI: Confidence interval
CT: Computer tomography
DFS: Disease-free survival
EAC: Esophageal adencarcinoma
EC: Esophageal cancer
ESCC: Esophageal squamous cell carcinoma
FBG: Fasting blood glucose
HR: Hazard ratio
NLR: Neutrophil to lymphocyte ratio
OS: Overall survival
PET: Positron emission tomography
REE: Resting energy expenditure
SCC: Squamous cell carcinoma

## References

[1] Kamangar F, Dores GM, Anderson WF (2006). Patterns of cancer incidence, mortality, and prevalence across five continents: defining priorities to reduce cancer disparities in different geographic regions of the world. J Clin Oncol.

[2] Jemal A, Bray F, Center MM, Ferlay J, Ward E, Forman D (2011). Global cancer statistics. CA Cancer J Clin.

[3] Harvie MN, Howell A, Thatcher N, Baildam A, Campbell I (2005). Energy balance in patients with advanced NSCLC, metastatic melanoma and metastatic breast cancer receiving chemotherapy-a longitudinal study. Br J Cancer.

[4] Smith M, Zhou M, Whitlock G, Yang G, Offer A, Hui G (2008). Esophageal cancer and body mass index: results from a prospective study of 220,000 men in China and a meta-analysis of published studies. Int J Cancer.

[5] Hayashi Y, Correa AM, Hofstetter WL, Vaporciyan AA, Rice DC, Walsh GL (2010). The influence of high body mass index on the prognosis of patients with esophageal cancer after surgery as primary therapy. Cancer.

[6] Yoon HH, Lewis MA, Shi Q, Khan M, Cassivi SD, Diasio RB (2011). Prognostic impact of body mass index stratified by smoking status in patients with esophageal adenocarcinoma. J Clin Oncol.

[7] Blom RL, Lagarde SM, Klinkenbijl JH, Busch OR, van Berge Henegouwen MI (2012). A high body mass index in esophageal cancer patients does not influence postoperative outcome or long-term survival. Ann Surg Oncol.

[8] Mifflin MD, St Jeor ST, Hill LA, Scott BJ, Daugherty SA, Koh YO (1990). A new predictive equation for resting energy expenditure in healthy individuals. Am J Clin Nutr.

[9] Rapp K, Schroeder J, Klenk J, Ulmer H, Concin H, Diem G (2006). Fasting blood glucose and cancer risk in a cohort of more than 140,000 adults in Austria. Diabetologia.

[10] LeRoith D, Novosyadlyy R, Gallagher EJ, Lann D, Vijayakumar A, Yakar S (2008). Obesity and type 2 diabetes are associated with an increased risk of developing cancer and a worse prognosis; epidemiological and mechanistic evidence. Exp Clin Endocrinol Diabetes.

[11] Nerlich AG, Hagedorn HG, Boheim M, Schleicher ED (1998). Patients with diabetes-induced microangiopathy show a reduced frequency of carcinomas. In Vivo.

[12] Diakos CI, Charles KA, McMillan DC, Clarke SJ (2014). Cancer-related inflammation and treatment effectiveness. Lancet Oncol.

[13] Proctor MJ, Morrison DS, Talwar D, Balmer SM, O’Reilly DS, Foulis AK (2011). An inflammation-based prognostic score (mGPS) predicts cancer survival independent of tumour site: a Glasgow Inflammation Outcome Study. Br J Cancer.

[14] McMillan DC (2013). The systemic inflammation-based Glasgow Prognostic Score: a decade of experience in patients with cancer. Cancer Treat Rev.

[15] Onodera T, Goseki N, Kosaki G (1984). Prognostic nutritional index in gastrointestinal surgery of malnourished cancer patients. Nihon Geka Gakkai Zasshi.

[16] McMillan DC, Watson WS, O’Gorman P, Preston T, Scott HR, McArdle CS (2001). Albumin concentrations are primarily determined by the body cell mass and the systemic inflammatory response in cancer patients with weight loss. Nutr Cancer.

[17] Templeton AJ, McNamara MG, SŠeruga B, Vera-Badillo FE, Aneja P, Ocaña A (2014). Prognostic role of neutrophil-to-lymphocyte ratio in solid tumors: a systematic review and meta-analysis. J Natl Cancer Inst.

[18] Han LH, Jia YB, Song QX, Wang JB, Wang NN, Cheng YF (2015). The prognostic significance of preoperative lymphocyte-monocyte ratio in patients with resectable esophageal squamous cell carcinoma. Asian Pac J Cancer Prev.

[19] Xie X, Luo KJ, Hu Y, Wang JY, Chen J. Prognostic value of preoperative platelet-lymphocyte and neutrophil-lymphocyte ratio in patients undergoing surgery for esophageal squamous cell cancer. Dis Esophagus. 2014;19. doi: 10.1111/dote.12296 10.1111/dote.12296 25410116

[20] Rashid F, Waraich N, Bhatti I, Saha S, Khan RN, Ahmed J (2010). A pre-operative elevated neutrophil: lymphocyte ratio does not predict survival from oesophageal cancer resection. World J Surg Oncol.

[21] Dutta S, Crumley AB, Fullarton GM, Horgan PG, McMillan DC (2011). Comparison of the prognostic value of tumour- and patient-related factors in patients undergoing potentially curative resection of oesophageal cancer. World J Surg.

[22] Guthrie GJ, Charles KA, Roxburgh CS, Horgan PG, McMillan DC, Clarke SJ (2013). The systemic inflammation-based neutrophil-lymphocyte ratio: experience in patients with cancer. Crit Rev Oncol Hematol.

[23] Grotenhuis BA, Wijnhoven BP, Hotte GJ, van der Stok EP, Tilanus HW, van Lanschot JJ (2010). Prognostic value of body mass index on short-term and long-term outcome after resection of esophageal cancer. World J Surg.

[24] Melis M, Weber JM, McLoughlin JM, Siegel EM, Hoffe S, Shridhar R (2011). An elevated body mass index does not reduce survival after esophagectomy for cancer. Ann Surg Oncol.

[25] Morgan MA, Lewis WG, Hopper AN, Escofet X, Harvard TJ, Brewster AE (2007). Prognostic significance of body mass indices for patients undergoing esophagectomy for cancer. Dis Esophagus.

[26] Rehman HU, Mohammed K (2003). Preoperative management of diabetic patients. Curr Surg.

[27] De Giorgio R, Barbara G, Cecconi A, Corinaldesi R, Mancini AM (2000). Diabetes is associated with longer survival rates in patients with malignant tumors. Arch Intern Med.

[28] Bartling B, Simm A, Sohst A, Silber RE, Hofmann HS (2011). Effect of diabetes mellitus on the outcome of patients with resected non-small cell lung carcinoma. Gerontology.

[29] Johnson G, Salle A, Lorimier G, Laccourreye L, Enon B, Blin V (2008). Cancer cachexia: measured and predicted resting energy expenditures for nutritional needs evaluation. Nutrition.

[30] Cannata D, Fierz Y, Vijayakumar A, LeRoith D (2010). Type 2 diabetes and cancer: what is the connection?. Mt Sinai J Med.

